# Short‐Term Glucocorticoid Treatment Reduces Circulating Sclerostin Concentrations in Healthy Young Men: A Randomized, Placebo‐Controlled, Double‐Blind Study

**DOI:** 10.1002/jbm4.10341

**Published:** 2020-06-13

**Authors:** Merel Jacobsson, Daniël H van Raalte, Annemieke C Heijboer, Martin den Heijer, Renate T de Jongh

**Affiliations:** ^1^ Department of Internal Medicine, Division of Endocrinology, Amsterdam UMC Amsterdam The Netherlands; ^2^ Endocrine Laboratory, Department of Clinical Chemistry, Amsterdam UMC University of Amsterdam Amsterdam The Netherlands

**Keywords:** BIOCHEMICAL MARKERS OF BONE TURNOVER, CLINICAL TRIALS, CORTICOSTEROIDS, SCLEROSTIN, Wnt

## Abstract

Glucocorticoid use is the most common cause of osteoporosis in young individuals. In the current study, we investigated the effects of glucocorticoid treatment on circulating sclerostin concentrations and serum bone turnover markers in healthy young men. We performed additional measurements in two combined randomized, placebo‐controlled, double‐blind, dose–response intervention studies: 64 healthy men (age: 22 ± 2 years; BMI: 22.1 ± 1.7 kg/m^2^) were allocated to receive placebo (*n* = 16), prednisolone 7.5 mg once daily (*n* = 24), or prednisolone 30 mg once daily (*n* = 24) for 2 weeks using block randomization. Primary outcome variables were serum sclerostin and serum bone turnover markers (CTx and P1NP), before and after the intervention. Baseline characteristics and variables did not differ between intervention groups. Compared with placebo, prednisolone high‐dose decreased serum sclerostin concentrations (−8.5 [−28.0 to 7.3] versus 1.5 [−6.5 to 20.0] pg/mL, *p* = 0.048), decreased P1NP concentrations (−28.0 [−39.3 to −18.3] versus –1.5 [−15.3 to 11.3] μg/L, *p* < 0.001) and increased CTx concentrations (108.0 [55.0 to 177.0] versus 64.0 [−24.3 to 120.0] ng/L, *p* = 0.038). Compared with placebo, prednisolone low‐dose did not alter sclerostin concentrations (*p* = 0.5) or CTx concentrations (*p =* 0.7), but tended to decrease P1NP concentrations (−9.0 [−24.0 to −1.3] versus –1.5 [−15.3 to 11.3] μg/L, *p* = 0.095). At baseline concentrations of sclerostin were positively correlated with concentrations of CTx (Spearman's rank correlation coefficient ρ = +0.409, *p* = 0.001), but not with P1NP. No significant correlations were observed between changes in outcome variables during the interventions. Short‐term high‐dose, but not low‐dose, prednisolone treatment reduces serum sclerostin concentrations in healthy young men. Whether this reflects a counter regulatory mechanism to compensate glucocorticoid‐induced negative effects through other mechanisms remains to be elucidated. © 2020 The Authors. *JBMR Plus* published by Wiley Periodicals, Inc. on behalf of American Society for Bone and Mineral Research.

## Introduction

Glucocorticoid use is the most common cause of secondary osteoporosis.^1^, ^2^ Even low doses of oral glucocorticoid administration such as 2.5 mg per day lead to a 1.5‐fold increase in fracture risk, whereas the relative risk rises up to fivefold for daily doses of 7.5 mg per day or higher in humans.^3^

The detrimental effect of glucocorticoids on bone is the net result of hampered new bone formation and an imbalance between bone formation and bone resorption favoring the latter.^4^ Glucocorticoids impair osteoblastic differentiation and maturation, while at the same time also inducing osteoblast apoptosis, which results in a decrease in number and function of osteoblasts.^4^, ^5^ In addition, osteoclast function and number is increased, in particular at the beginning of glucocorticoid exposure. This is the result of both direct effects on apoptotic signaling of osteoclasts and indirect effects through increased RANKL (receptor activator of NF‐κB ligand) production by osteoblasts and reduced expression of osteoprotegerin (OPG), the RANKL decoy receptor.^4^

One of the potential mechanisms contributing to the detrimental effects of glucocorticoid exposure on bone is inhibition of the Wnt/β‐catenin signaling pathway. The Wnt/β‐catenin signaling pathway drives the differentiation of stem cells to the osteoblast lineage and supports differentiation and survival of osteoblasts. Furthermore, the Wnt/β‐catenin signaling pathway inhibits osteoclast function and number through the increased expression of OPG rather than RANKL by the osteoblast.^1^, ^2^, ^5^, ^6^, ^7^, ^8^, ^9^ Sclerostin, a glycoprotein produced by osteocytes primarily, is an antagonist of the Wnt/β‐catenin signaling pathway and seems to regulate the number and activity of osteoblasts.^10^

In mice, glucocorticoids increased the expression of Sost, the gene encoding sclerostin production.^11^ In female KO mice lacking Sost and thus characterized by activation of Wnt/β‐catenin signaling, bone mass and strength were maintained in conditions of glucocorticoid excess.^11^ Moreover, antibodies against sclerostin in mice prevented glucocorticoid‐induced osteoporosis and appeared to maintain osteoblast activity.^12^

In accordance with the rodent studies, in patients treated with glucocorticoids for 12 months (7.5 mg/day), serum sclerostin concentrations showed a significant increase. Furthermore, as expected, a decrease in the bone formation marker P1NP and an increase in the bone resorption marker CTx were found.^13^ In contrast, in other studies glucocorticoids seem to have the opposite action on serum sclerostin concentrations. In one study, patients with various medical pathologies requiring glucocorticoid therapy (10 to 50 mg per day) were included. After 96 hours of treatment, a significant reduction of serum sclerostin was observed.^14^ A time‐dependent effect is supported by a recent study in patients with autoimmune diseases treated with glucocorticoids (30 to 60 mg per day). After 1 week, circulating Wnt signaling inhibitors increased, but from the second week onward they decreased.^15^ Also, patients with chronic endogenous hypercortisolism demonstrated decreased serum sclerostin concentrations.^16^

Taken together, human data on the effects of glucocorticoid treatment on circulating sclerostin concentrations and bone turnover markers are scarce and have shown contradictory results. These contradictory results may be explained by the difference in assays used to measure sclerostin, the heterogeneity of the included patients, the different dosages of the used prednisolone, and the difference in follow‐up time. Another main reason for contradictory results is the variability in inflammatory diseases as an indication for glucocorticoid treatment, which may also have directly influenced bone metabolism.

Furthermore, the studies were not set‐up in a blinded or randomized fashion. Therefore, the aim of this study was to examine the effects of short‐term low‐ and high‐dose glucocorticoid treatment on circulating sclerostin concentrations and serum bone turnover markers in healthy individuals.

## Materials and Methods

### Participants

For the current study, additional measurements of participants in two combined randomized controlled trials were done.^17^, ^18^ For both these studies, 32 (total *n* = 64) healthy white men were recruited by local advertisements. Inclusion criteria were age 18 to 35 years, BMI 20.0 to 25.0 kg/m^2^, and good physical health (determined by medical history, physical examination, and screening blood tests). Exclusion criteria were smoking, shift work, any current illness, use of any medication, a history of glucocorticoid use, excessive sports activity (ie, more often than twice a week), and recent changes in weight or physical activity. All participants provided written informed consent before participation. The original studies were approved by an independent ethics committee and were conducted in accordance with the Declaration of Helsinki.

### Study design

For the current study, data were used from two combined randomized, placebo‐controlled, double‐blind, dose–response intervention studies.^17^, ^18^ Following assessment of eligibility and baseline measurements, participants were randomized to receive placebo (*n* = 16), prednisolone 7.5 mg once daily (*n* = 24), or prednisolone 30 mg once daily (*n* = 24) for 2 weeks using block randomization, as carried out by the Department of Experimental Pharmacology of the VU University Medical Center (Amsterdam, The Netherlands). Before treatment and on day 13 of treatment, blood was drawn in the fasted state and serum was stored at −80°C for measurement of serum sclerostin concentrations and bone turnover markers, P1NP and CTx.

### Blood pressure measurement

Blood pressure (systolic and diastolic) were manually measured (Welch Allyn, Delft, The Netherlands) by a single experienced investigator following the 30‐min acclimatization period. The average of three consecutive blood pressure measurements was used.

### Study medication

Placebo tablets were obtained from Xendo Drug Development (Groningen, The Netherlands) and prednisolone (7.5 mg and 30 mg) tablets were purchased from Pfizer (Sollentuna, Sweden). To blind the treatment, tablets were encapsulated.

### Biochemical analyses

Concentrations of circulating sclerostin were measured by an electrochemiluminescence assay (MSD 96‐well MULTI‐ARRAY Human Sclerostin Assay; Meso Scale Discovery/Meso Scale Technologies, Rockville, MD, USA) with an intraassay coefficient of variation (CV) ≤6.0%. Interassay CV was 11% and the lower limit of quantitation was 0.040 ng/mL. Electrochemiluminescence immunoassays (Cobas 6000; Roche Diagnostics, Almere, The Netherlands) were used to measure serum P1NP with an interassay CV of <8.5% and serum CTx with an interassay CV of <5.5%.

### Statistical analyses

Data are presented as means ± SD or as medians (interquartile range) in case of skewed distribution. Differences in baseline characteristics between treatment groups were evaluated using ANOVA or the Kruskal–Wallis test. Absolute changes from baseline were calculated (posttreatment value minus pretreatment value). To evaluate treatment‐induced differences in serum sclerostin, P1NP, and CTx concentrations, Wilcoxon signed‐rank tests were performed. To analyze differences in treatment‐induced effects between intervention groups, Kruskal–Wallis tests with trend analysis (Jonckheere–Terpstra tests) were performed. In case of relevant differences between intervention groups, post hoc testing using the Wilcoxon rank sum test (Mann–Whitney *U* test) was performed. Correlations between variables were assessed with Spearman correlations. Statistical analyses were run using IBM SPSS Statistics for Windows version 22.0 (SPSS, Chicago, IL, USA); *p* < 0.05 was considered statistically significant. Graphs were made using GraphPad Prism for Windows version 8.0.2 (GraphPad Software, La Jolla, CA, USA).

## Results

### Baseline characteristics

Before treatment, all treatment groups had comparable age, anthropometrics, and blood pressure. Baseline concentrations of circulating sclerostin, P1NP, and CTx did not differ between treatment groups (Table 1).

**Table 1:** Baseline Characteristics and Variables

	Placebo	Prednisolone 7.5 mg	Prednisolone 30 mg	*p*
*n*	16	24	24	
Age (years)	22 ± 3	22 ± 2	22 ± 2	0.982
Weight (kg)	77 ± 8	75 ± 6	75 ± 8	0.800
Height (cm)	185 ± 6	184 ± 7	185 ± 4	0.659
BMI (kg/m^2^)	22.3 ± 1.8	22.3 ± 1.7	21.9 ± 1.8	0.642
SBP (mm Hg)	120 ± 9	124 ± 8	121 ± 11	0.461
DBP (mm Hg)	75 ± 11	73 ± 11	77 ± 12	0.445
Sclerostin (pg/mL)	98 [85 to 114]	115 [91 to 132]	108 [86 to 124]	0.135
P1NP (μg/L)	69 [60 to 115]	82 [60 to 103]	72 [55 to 101]	0.555
CTx (ng/L)	574 [422 to 758]	606 [478 to 746]	541 [433 to 650]	0.557

Data are means ± SD or as medians [interquartile range]. Between‐group baseline differences were tested by ANOVA or by Kruskal‐Wallis test as non‐parametric test (*p*).

SBP = systolic blood pressure; BDP = diastolic blood pressure.

### Treatment‐induced effects

Treatment with prednisolone 30 mg once daily decreased concentrations of sclerostin (*p* = 0.033; Table 2). Furthermore, treatment with prednisolone 30 mg once daily also decreased serum P1NP (*p* < 0.001) and increased serum CTx (*p* = 0.002; Table 2). Treatment with prednisolone 7.5 mg once daily did not alter concentrations of sclerostin and CTx (*p* = 0.637 and *p* = 0.168, respectively). However, treatment with prednisolone 7.5 mg once daily decreased serum P1NP (*p* = 0.002; Table 2). Administration of placebo did not alter concentrations of sclerostin, P1NP, and CTx (*p* = 0.509, *p* = 0.569, and *p* = 0.198, respectively; Table 2).

**Table 2:** Absolute changes from baseline in serum sclerostin, P1NP and CTx stratified for each intervention group

	Placebo		Prednisolone 7.5 mg		Prednisolone 30 mg	
	Change	*p*	Change	*p*	Change	*p*
Serum sclerostin (pg/mL)	1.5 [−6.5 to 20] (+1.5%)	0.509	‐1 [−26.25 to 16.5] (−0.9%)	0.637	−8.5 [−28 to 7.25] (−7.9%)	0.033
Serum P1NP (μg/L)	−1.5 [−15.25 to 11.25] (−2.2%)	0.569	−9 [−24 to −1.25] (−11.0%)	0.002	−28 [−39.25 to −18.25] (−38.9%)	0.001
Serum CTx (ng/L)	64 [−24.25 to 120] (+11.1%)	0.198	82 [−54.5 to 139.8] (+13.5%)	0.168	108 [55 to 177] (+20.0%)	0.002

Data represent absolute change from baseline expressed as medians (interquartile range) and percentages.

*p*, after treatment versus baseline within each intervention group.

### Differences in treatment‐induced absolute changes

**Figure 1:**
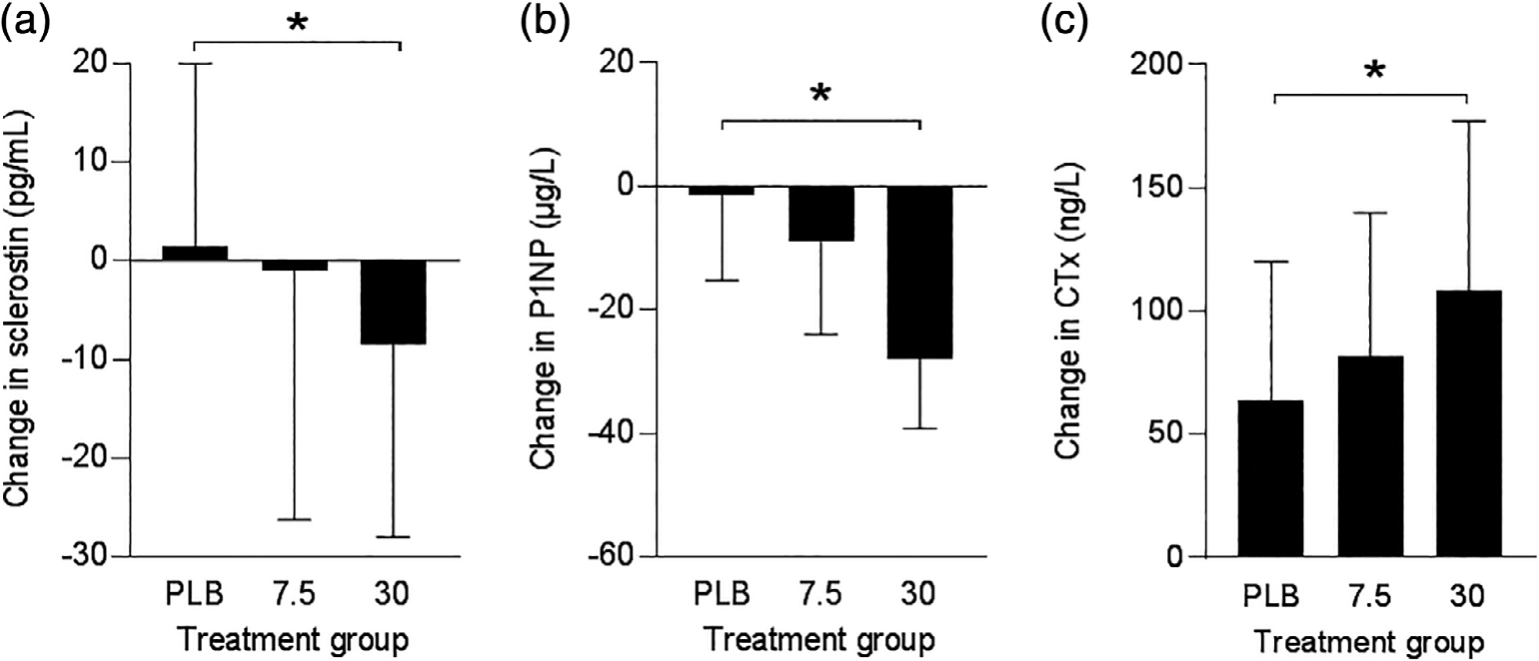
Change in serum sclerostin (*A*), P1NP (*B*), and CTx (*C*) concentrations during treatment. Data represent absolute change from baseline (medians [interquartile range]). PLB = placebo, 7.5 = prednisolone 7.5 mg once daily, and 30 = prednisolone 30 mg once daily group. *p < 0.05.

Between‐group changes from baseline were tested by Kruskal–Wallis test with trend analysis (the Jonckheere–Terpstra test). The absolute changes of serum sclerostin during treatment tended to be different between the intervention groups (*p* = 0.078; Fig. 1 *A*). The decrease in serum sclerostin in the prednisolone 30 mg once daily group, but not in the 7.5 mg once daily group, was significantly different from the change in the placebo group (Table 2; Fig. 1 *A*). Furthermore, the absolute changes in serum P1NP were different between treatment groups (*p <* 0.001; Fig. 1 *B*). The decrease in serum P1NP in the prednisolone 30 mg once daily, but not in the 7.5 mg once daily group, was significantly different from the change in the placebo group (Table 2; Fig. 1 *B*). The increased serum CTx during treatment tended to be different between the intervention groups (*p* = 0.064; Fig. 1 *C*). The increase in serum CTx in the prednisolone 30 mg once daily group, but not the 7.5 mg once daily group, was significantly different from the increase in the placebo group (Table 2; Fig. 1 *C*).

### Correlations

**Figure 2:**
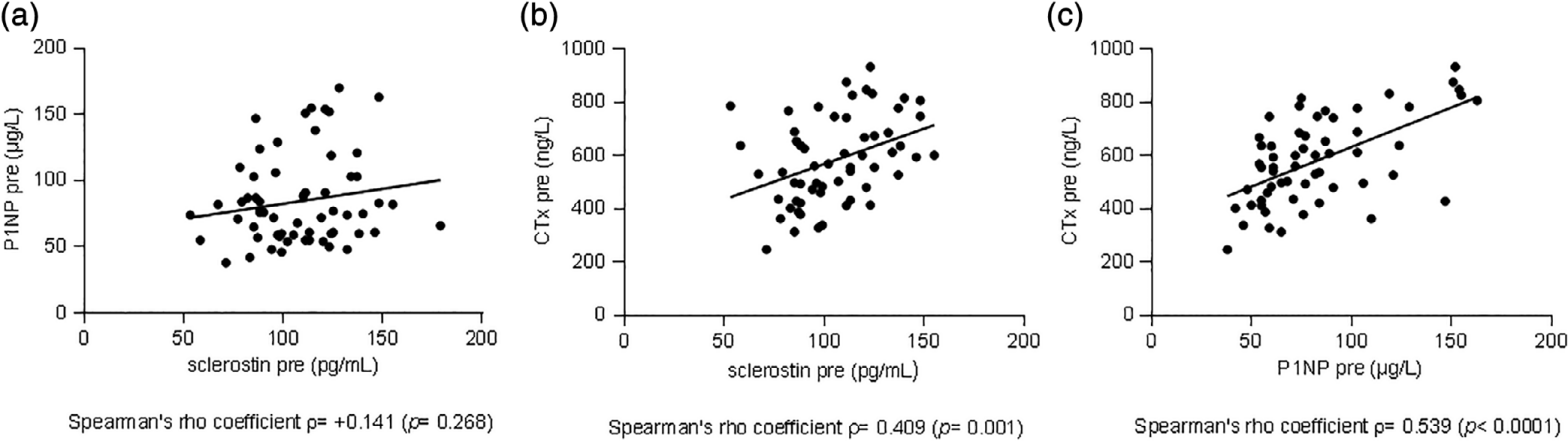
Correlations between baseline concentrations of sclerostin and P1NP (a), sclerostin and CTx (b) and P1NP and CTx (c).

At baseline, the serum CTx concentration was positively correlated with serum sclerostin concentration (*p* = 0.001; Fig. 2 *B*). Moreover, serum CTx concentration was positively correlated with serum P1NP concentration (*p* < 0.001; Fig. 2 *C*). At baseline, no significant correlation was found between serum P1NP and sclerostin concentrations (*p* = 0.268; Fig. 2 *A*). No significant correlations were observed between change in P1NP and change in sclerostin concentration (*p* = 0.629), between change in CTx and change in sclerostin concentration (*p* = 0.975), or between change in CTx and change in P1NP concentration (*p* = 0.331) in all treatment groups together. In addition, for each treatment group separately no significant correlations were observed (data not shown).

### Safety and tolerability

According to the original studies,^17^, ^18^ one participant receiving prednisolone 30 mg once daily reported mild and transient sleeplessness during treatment; one other participant in the prednisolone 30 mg once daily group reported mild gastric discomfort. No other side effects were reported in either group.

## Discussion

This is the first study about the effects of low‐ and high‐dose prednisolone treatment on serum sclerostin in healthy young men in the setting of a randomized placebo‐controlled, double‐blind trial. Our study showed that both low‐ and high‐dose prednisolone decreased concentrations of the bone formation marker P1NP and that only the high‐dose treatment also increased concentrations of the bone resorption marker CTx. This double effect on bone metabolism may imply glucocorticoid‐induced inhibition of the Wnt/β‐catenin signaling pathway. However, short‐term high‐dose prednisolone treatment decreased concentrations of the Wnt signaling inhibitor sclerostin, whereas low‐dose prednisolone had no significant effects.

Based on studies in vitro and in rodents,^1^, ^2^, ^4^, ^14^ it is thought that the main action by which glucocorticoids impair osteoblastic differentiation is by increasing activity of sclerostin, an inhibitor of the Wnt/β‐catenin signaling pathway. However, the results of the present study do not support this hypothesis. High‐dose prednisolone treatment reduced serum sclerostin concentrations in our study. This finding is in agreement with most^14^, ^15^, ^16^ but not all previous studies^13^ after effects of glucocorticoid exposure on circulating sclerostin levels in various patient populations.^16^ A possible explanation for reduced sclerostin concentrations could be that prednisolone induces osteocyte apoptosis and thus a reduction in sclerostin‐producing osteocytes that overshadows potential stimulating effects on sclerostin production. Previous studies have shown that bone of patients using glucocorticoids is characterized by osteocyte apoptosis.^19^, ^20^ Also, in mice, glucocorticoid exposure induced osteocyte apoptosis.^20^, ^21^ This underlying mechanism is supported by the finding that in patients with endogenous hypercortisolism after treatment, an increased serum FGF23 was found, which is considered as an independent marker of well‐functioning osteocytes.^22^ Unfortunately, we were not able to measure FGF23 concentrations in our study samples. Another explanation may be that the reduction in sclerostin concentrations could represent only a compensatory mechanism to counteract the inhibition of the Wnt pathway induced by glucocorticoids.^9^ In that case, glucocorticoids may inhibit the Wnt/β‐catenin signaling pathway through other mediators such as DKK‐1, bone morphogenetic proteins, or hydrogen sulfide.^23^, ^24^ However, part of the glucocorticoid effects on osteoblasts may be independent of the Wnt/β‐catenin signaling pathway. For example, glucocorticoids directly induce osteoblast apoptosis through activation of caspase 3.^1^, ^25^ However, glucocorticoids not only suppress bone formation, but also increase bone resorption by an increase in osteoclast function and number.^4^ The Wnt/β‐catenin signaling pathway inhibits osteoclast function and number through inducing an imbalance in the RANKL–OPG ratio.^1^, ^2^, ^5^ A decrease in sclerostin concentrations as we found in our study could be considered an attempt to compensate the detrimental effects of glucocorticoids on bone either through the Wnt/β‐catenin signaling pathway or through other mechanisms.

In our study, high‐dose, but not low‐dose, glucocorticoid treatment decreased sclerostin concentrations. Although one may speculate that the lack of power may have resulted in a nonsignificant finding in the low‐dose treatment group, we consider this unlikely as the point estimate of change in serum sclerostin concentrations in the low‐dose group was very close to zero. A potential threshold in the amount of glucocorticoid exposure to decrease sclerostin concentrations is in line with previous findings. High dosages of prednisolone (a mean dose of 64 ± 16 mg per day) prescribed in patients for several indications induced a significant reduction of serum sclerostin concentrations after 96 hours.^14^ Another study in patients prescribed glucocorticoids (30 to 60 mg per day) for autoimmune disease demonstrated a decrease in circulation Wnt signaling inhibitors, but only after 2 weeks or more.^15^ A lower glucocorticoid dosage (prednisolone equivalent dose of 23.1 ± 12.7 mg per day) did not change serum sclerostin concentrations in hematology patients as compared with controls after nearly 50 days and even increased serum sclerostin concentrations at 12 months.^13^ In the latter study, it is shown that duration of treatment also mediates the glucocorticoid‐induced effect. Unfortunately, to the best of our knowledge, there are no studies regarding the time sequence of glucocorticoid‐induced effects on osteocyte number and function.

In agreement with previous studies in humans,^13^, ^14^, ^26^ the current study shows a glucocorticoid‐induced decrease in serum P1NP and an increase in serum CTx concentrations. Furthermore, the administration of glucocorticoids demonstrated a clear dose dependency in these effects. The combination of slightly reduced sclerostin concentrations and also reduced P1NP levels implicates that the true net stimulating effect on osteoblasts and thus the clinical significance of the glucocorticoid‐induced change in sclerostin concentrations may be limited.

In the present study, at baseline a significant positive correlation was found between serum sclerostin with CTx, but not with P1NP concentrations. Results in other studies regarding correlations between sclerostin concentrations and concentrations of bone turnover markers are contrasting.^27^, ^28^, ^29^ Sclerostin is thought to act predominantly as an inhibitor of bone formation. However, in mice, the Wnt/β‐catenin signaling pathway predominantly anabolic for bone, is switched to anticatabolic in situations of glucocorticoid excess.^11^ Furthermore, in human primary preosteocyte cultures and mouse osteocyte‐like cells, recombinant human sclerostin administration results in a stimulation of osteoclast activity by a RANKL‐mediated mechanism.^30^ Thus, the association between serum sclerostin and CTx concentrations in our study may also indicate a potential effect of sclerostin on bone resorption. The absence of correlations between treatment‐induced changes in outcomes may underline that glucocorticoid effects on bone cells are complex and probably multifactorial.

An important strength of our study is that it is the first randomized, controlled, and double‐blinded study on this subject. Another strength is that our study is performed in healthy young men instead of patients with an indication for glucocorticoid treatment. In particular, chronic inflammatory diseases alter osteocyte function and cause osteocyte apoptosis, and may thus interfere with glucocorticoid‐induced effects.^31^ The use of a healthy young population of males only, however, does also limit the applicability of our findings to daily practice. Another limitation is that this study combines two equally designed placebo‐controlled, double‐blind, dose–response intervention trials that were not designed to study sclerostin or bone turnover markers as outcome variables, neither powered for this purpose. Nevertheless, we were able to demonstrate clear dose–response relationships for the bone turnover markers and significant changes in the high‐dose treatment group. A further limitation is that sclerostin concentrations were measured in the circulation and not at the level of the bone tissue itself. However, measurements in bone tissue require invasive procedures that were not feasible to perform in the applied study protocols; a previous study demonstrated a strong correlation between circulating and bone marrow sclerostin concentrations.^32^ Furthermore, blood sampling was only performed at two time points, which hampered any study of glucocorticoid‐induced sclerostin kinetics and other markers of the Wnt signaling pathway were not analyzed such as DKK1. Finally, it should be noted that differences in assays used to measure sclerostin and medium in which it is measured, ie, serum or plasma, in different studies may partly explain the variability in findings.^33^, ^34^, ^35^

An excess of glucocorticoids, either endogenously or iatrogenically induced, leads to loss of bone and is one of the leading causes of increased bone fragility worldwide.^36^ Of patients on chronic prednisolone treatment, 30% to 50% sustain a fracture,^2^, ^22^ with accompanying healthcare costs per person and for society. The present study suggests that short‐term high doses, not low doses, of prednisolone decreased serum sclerostin concentrations in healthy men. We speculate that this may have consequences for the applicability of sclerostin antibodies in the treatment of glucocorticoid‐induced osteoporosis. It seems plausible that the decrease in sclerostin concentrations is a counterregulatory attempt to oppose the negative effects of inhibition of Wnt signaling induced by glucocorticoids through other mechanisms, although this hypothesis remains to be proven. Another explanation for the decrease in sclerostin concentrations may be a direct effect of glucocorticoids on osteocyte survival. Larger randomized controlled trials after glucocorticoid exposure of different durations in healthy individuals, both men and women of all ages, are necessary to confirm our results—preferably with bone biopsies to study effects at the bone‐tissue level.
